# Complete Genome Sequence of Salmonella enterica Serovar Enteritidis NCM 61, with High Potential for Biofilm Formation, Isolated from Meat-Related Sources

**DOI:** 10.1128/MRA.01434-18

**Published:** 2019-01-10

**Authors:** Huhu Wang, Linlin Cai, Haijing Hu, Xinglian Xu, Guanghong Zhou

**Affiliations:** a Key Lab of Meat Processing and Quality Control, Ministry of Education, Nanjing Agricultural University, Nanjing, People’s Republic of China; b MOE Joint International Research Laboratory of Animal Health and Food Safety, Nanjing Agricultural University, Nanjing, People’s Republic of China; University of Arizona

## Abstract

Here, we report the complete genome sequence of strain NMC 61 of Salmonella enterica serovar Enteritidis, which was previously isolated from conveyor belts during chicken slaughter and has the potential to form biofilms on several surfaces. The genome is predicted to contain 110 noncoding small RNAs on the chromosome.

## ANNOUNCEMENT

Salmonella belonging to Enterobacteriaceae is a globally widespread foodborne pathogen. All are at risk for Salmonella infection but especially those with weakened immune systems. Salmonella enterica serovar Enteritidis is the most frequently detected causative agent in foodborne outbreaks and recalls (1, 2). Complete elimination of Salmonella bacteria is often challenging due to the formation of protective biofilms, which is significantly affected by intracellular and environmental factors (3–5) and can vary based on the source of isolation. Biofilms present on conveyor belts can serve as sources of contamination for meat during processing (6). However, the many strains that have had their genomes completely sequenced have been isolated from sources outside those involved in meat processing (7, 8). Therefore, the complete genome sequence of S. Enteritidis strain NCM 61, which was isolated from the conveyor belts of chicken by wiping, and had a high potential for biofilm formation (9, 10), was determined in this study.

A single colony of S. Enteritidis NCM 61 was picked to 5 ml of Trypticase soy broth (TSB) and incubated at 37°C for 18 h with shaking. The DNA was extracted using a QIAamp DNA microbiome kit and used to prepare a library with a fragment size of >10 Kb selected with the BluePippin system. The genome of S. Enteritidis was sequenced on a Pacific Biosciences instrument (PacBio, Menlo Park, CA) using P4-C2 chemistry. Hierarchical Genome Assembly Process (HGAP) software (version 2.3.0) was used to assemble a total of 86,659 reads with an average length of 11,156 bp. The *de novo* assembled chromosome genome of S. Enteritidis NCM 61 is 4,679,739 bp with GC content of 52.17%. The 4,660 coding sequences, 106 structural RNAs (22 rRNAs and 84 tRNAs), and 14 gene islands (GIs) were annotated using Glimmer version 1.1, rRNAmmer 1.2 server, tRNAscan-SE 2.0, Rfam 14.0, and IslandViewer 4 (11, 12). One 59,387-bp circular plasmid (GC content of 51.92% and 109 predicted coding regions) was also identified during assembly by HGAP. A total of 380 genes on the chromosome were predicted to be associated with bacterial virulence factors, and 16 multiresistance determinants were found on the chromosome using ardbAnno version 1.0 software (13), including *arc*, *mdt*, *tol*, *pbp*, *mdf*, *mac*, *ksg*, *emr*, *bcr*, *arn*, and *acr*. The chromosome genome collinearities between the NCM 61 strain and S. Typhimurium LT2 (GenBank accession number AE006468), S. Heidelberg SL476 (CP001120), and S. Enteritidis Durban (CP007507) were 95.04%/91.54%, 94.18%/90.14%, and 99.12%/99.14%, respectively, which were determined using NCBI BLAST between two genomes with an E value less than or equal to 10^−5^ and alignments exceeding 80% identity within 1,000 bp of the query sequence. In addition, using the Rfam database, a total of 110 noncoding small RNAs were identified on the chromosome. The availability of the whole-genome sequence will provide future insight into the adhesion and pathogenic profiles of the S. Enteritidis NCM 61 strain.

### Data availability.

The whole-genome sequence for Salmonella Enteritidis strain NCM 61 has been deposited in NCBI under the BioProject accession number PRJNA492709. The GenBank accession number is CP032851 for the chromosome and CP032850 for the plasmid. The SRA accession number is SRS3887548.
